# Gazing into Thin Air: The Dual-Task Costs of Movement Planning and Execution during Adaptive Gait

**DOI:** 10.1371/journal.pone.0166063

**Published:** 2016-11-08

**Authors:** Toby J. Ellmers, Adam J. Cocks, Michail Doumas, A. Mark Williams, William R. Young

**Affiliations:** 1 Department of Life Sciences, Brunel University London, Uxbridge, United Kingdom; 2 School of Psychology, Queen’s University Belfast, Belfast, United Kingdom; 3 College of Heath, University of Utah, Salt Lake City, Utah, United States of America; 4 Centre for Cognitive Neuroscience, College of Health and Life Sciences, Brunel University London, Uxbridge, United Kingdom; 5 Institute of Environment, Health and Societies, Brunel University London, Uxbridge, United Kingdom; Center for BrainHealth, University of Texas at Dallas, UNITED STATES

## Abstract

We examined the effect of increased cognitive load on visual search behavior and measures of gait performance during locomotion. Also, we investigated how personality traits, specifically the propensity to consciously control or monitor movements (trait movement ‘reinvestment’), impacted the ability to maintain effective gaze under conditions of cognitive load. Healthy young adults traversed a novel adaptive walking path while performing a secondary serial subtraction task. Performance was assessed using correct responses to the cognitive task, gaze behavior, stepping accuracy, and time to complete the walking task. When walking while simultaneously carrying out the secondary serial subtraction task, participants visually fixated on task-irrelevant areas ‘outside’ the walking path more often and for longer durations of time, and fixated on task-relevant areas ‘inside’ the walkway for shorter durations. These changes were most pronounced in high-trait-reinvesters. We speculate that reinvestment-related processes placed an additional cognitive demand upon working memory. These increased task-irrelevant ‘outside’ fixations were accompanied by slower completion rates on the walking task and greater gross stepping errors. Findings suggest that attention is important for the maintenance of effective gaze behaviors, supporting previous claims that the maladaptive changes in visual search observed in high-risk older adults may be a consequence of inefficiencies in attentional processing. Identifying the underlying attentional processes that disrupt effective gaze behaviour during locomotion is an essential step in the development of rehabilitation, with this information allowing for the emergence of interventions that reduce the risk of falling.

## Introduction

As we walk through our cluttered world, we rely predominantly on visual information. An effective visual search strategy allows for the identification of hazards and for the feedforward planning and execution of safe stepping behaviors to avoid tripping. The decline in visual function with age is a well-established and an independent predictor of increased fall-risk [1–3]. Falls in older adults are the leading cause of injury and mortality in those aged 65 years and over [4,5]. In 2013, the annual health costs from fall-related injury in the UK exceeded £2.3 billion [6], with this figure approaching $34 billion in the U.S. [7]. The negative impact that falls have on both the individual and the economy emphasises the need to identify factors that compromise stepping safety and to develop low-cost and effective interventions to reduce the risk of falls.

Stepping and eye movements exhibit a robust spatiotemporal relationship in healthy younger adults [8–10], with this relationship being disrupted during the ageing process [11–14]. In general, when navigating a series of stepping constraints younger adults fixate on a target momentarily prior to initiating the step towards it, with their gaze maintained on this target until the step has been completed [11,12]. However, older adults, particularly those deemed to be at a high risk of falling and/or those with higher levels of fall-related anxiety, transfer their gaze away from the target prior to the step being completed [11–14]. In addition, Young and colleagues [13] found that when approaching a target followed by a series of obstacles, low-risk older adults displayed a ‘proactive’ pattern of visual search, fixating on, and transferring their gaze between, these subsequent stepping constraints. However, high-risk older adults spent less time previewing these subsequent constraints and instead spent more time fixating the initial target. Adopting this less-variable pattern of visual search behavior, whereby the individual focuses on the initial target and fails to fixate on future constraints, is likely to compromise an individual’s ability to generate a ‘spatial map’ of their environment [15]. As a result, it has been suggested that the early transfer of gaze away from the target (i.e., prior to the step being completed) may be a consequence of high-risk older adults failing to pick up this visual information during the initial approach [16]. Given that this early transfer of gaze is causally linked to reduced stepping accuracy and a higher incidence of stepping errors [17], there is a clear need to identify the underlying mechanisms mediating this apparently maladaptive visual search strategy.

It has been suggested that the altered visual search behavior observed in high-risk older adults may be caused by inefficiencies in attentional processing [16]. It is well established that maintaining effective, safe locomotion requires cognitive input [18,19]. Cognitive impairment is associated with increased fall-risk in older adults [20] and walking while carrying out a secondary task negatively impacts measures of gait performance in both young [21,22] and older adults [23–26]. However, this dual-task interference, or dual-task costs (DTCs), appear to be most pronounced in high-risk older adults [27–29]. Furthermore, the inability to maintain locomotion while holding a conversation and instead ‘stopping walking when talking’ is a reliable predictor of older adult fall-risk [30]. These findings indicate that the ability to allocate attention efficiently between locomotion and a concurrent task may be impaired in high-risk older adults. It has also been suggested that the less-variable pattern of visual search behavior observed in high-risk (and high-anxious) older adults may be a consequence of these individuals not possessing sufficient cognitive resources to store a ‘spatial map’ of their surroundings [16]. However, to our knowledge no researchers have looked to investigate how increased cognitive load impacts visual search behavior during locomotion.

Psychological factors also influence attentional processing during gait. Under certain conditions, such as injury or accident (including older adult falls; [31]), performers may attempt to consciously monitor and control movements that are usually considered automatic (such as locomotion), often leading to a disruption in performance (i.e., [32]). This phenomenon is frequently described as ‘reinvestment’, with an individual’s propensity to consciously monitor and control their movements argued to be a dimension of personality (for a review, see [33]). In addition to disrupting the automaticity of motor performance, researchers have indicated that reinvestment can influence the allocation of attention during gait. Much in the same way as performing a cognitive dual-task while walking can reduce an individual's ability to perceive environmental cues [34,35], researchers have demonstrated that conscious monitoring and control of movement may similarly impair the perception of external information during locomotion [36,37]. Masters and colleagues [33] argued that cognitive resources are required to consciously attend to the process of moving. This process in turn limits the resources available for other processes, which may include the visual search necessary to attend to information in the external environment [16]. It has also been suggested that an internal focus of attention may impair the retention of an environmental ‘spatial map’ [16], with older adults who ‘stop walking when talking’ displaying both greater propensity to consciously control their movements and poorer retention of visuo-spatial information [37]. Yet, little is known about how reinvestment influences visual search behavior.

The primary aim in this present research was to investigate how an increased cognitive load influences visual search during adaptive gait. A healthy younger cohort was selected as the most appropriate sample to investigate this research question, so as to avoid any potential confounding age-related factors, such as cognitive decline. Furthermore, examining the effects of such a manipulation on younger adults allows for the identification of ‘healthy’ behaviors to later compare against a high-risk older adult cohort. Therefore, we examined whether the previously detailed less-variable patterns of visual search behaviors observed in high-risk older adults can be induced in a healthy younger adult cohort walking while simultaneously carrying out a cognitive dual-task. The secondary aim in this present research was to evaluate if participants with higher scores on a measure of trait-reinvestment exhibited reduced ability to visually preview the environment during locomotion.

We predicted that individuals would be less likely to proactively scan their environment and instead fixate for longer durations on single points during the walkway when concurrently processing a cognitive dual-task (mental arithmetic). Therefore, we expected a reduction in both the number of task-relevant ‘inside’ fixations (those within the walking path) while walking under cognitive load. As literature demonstrates how a propensity to consciously control movements is associated with a reduction in the ability to allocate attention between concurrent tasks during locomotion [36,37], we predicted that changes in visual search behavior under cognitive load would be most pronounced in high-trait-reinvesters. Finally, researchers have demonstrated that walking while simultaneously performing a cognitive dual-task can impair performance in both the motor- and cognitive-task in younger adults, relative to a single-task baseline condition [24]. Therefore, we predicted significant DTCs for both the motor- and cognitive-task. As consciously attending to the process of movement requires cognitive resources and may limit the resources available for other processes [33], we predicted these DTCs to be greatest in high-trait-reinvesters.

## Methods

### Participants

Fourteen young adults (male/female: 9/5; mean ± SD age: 26.36 ± 2.59 years) were recruited from undergraduate and postgraduate courses at the lead institution. Participants were free from any musculoskeletal or neurological impairment. Participants requiring the use of eyeglasses for daily locomotor activities were excluded due to incompatibility with the gaze tracking equipment. However, the use of contact lenses was permitted.

### Ethics Statement

Ethical approval was obtained by the local ethics committee at the lead institution and the research protocol was carried out in accordance with the principals laid down by the Declaration of Helsinki. All participants provided written and informed consent.

### Procedure

On arrival, participants were fitted with reflective markers placed on the sternum and mid-foot of both feet [38], and then with a Mobile Eye-XG portable eye-tracking system (ASL, Bedford, MA). The eye-tracking system records participants’ gaze by contrasting the pupil and corneal reflection, allowing the superimposition of a point of gaze crosshair on a video of the environment recorded from a scene camera. Once calibration was complete, gaze data were recorded wirelessly at 30 Hz using Eye Vision Software (ASL). The eye-tracker features an integrated microphone, which was used to record audio (also at 30 Hz). Kinematic data were collected at 150 Hz using an eight camera Motion Analysis system (MotionAnalysis, Santa Rosa, California).

**Fig 1:**
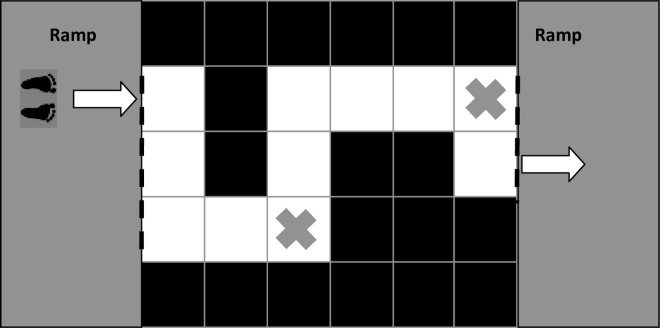
Schematic of an example path and direction of walking. The arrows indicate the route that participants took (returning on the left side of the walkway in all trials). Blocks marked with an ‘X’ were participants’ precision stepping targets. Dotted black lines indicate the points between which the time to complete the walking task was calculated.

The experimental set up described below is highly comparable to that previously used by Young and colleagues [37]. Participants walked at a comfortable pace over a 6 by 5 grid of 19 black and 11 white wooden blocks (stepping surface of each block = 40cm x 40cm, height of each block = 30cm, total length of the walking path = 4.4m). The white blocks were arranged to form one-of-four different non-linear routes that participants were instructed to traverse at a comfortable pace, without stepping on the black blocks (Fig 1). Each non-linear pattern contained a different combination of straight sections and two left and two right apexes. Two white blocks on each pattern were marked with an ‘X’ (the fifth and the tenth block of each pattern). These formed participants’ precision stepping targets. Participants were instructed to step on the middle of the ‘X’ with the middle of their foot (i.e., place the mid-foot markers of their swing foot as close to the centre of the target as possible). The protocol was designed to mimic the common task of walking on a pavement, targeting paving stones perceived to be stable and safe.

At the start of each trial, participants stood behind a 2.3 metre-high screen (preventing them from seeing the walkway). When instructed to “Go”, participants walked around the screen, up a ramp (120cm long), along the white blocks, and down a second ramp. They then walked off the ramp to the left side and returned behind the screen in anticipation of starting the subsequent trial.

Participants completed walks under two conditions of Cognitive Load: Baseline (no cognitive load) and Dual-Task. The Dual-Task condition consisted of walking while concurrently subtracting out loud from a randomized number (between 90 and 110) in 7s. Participants were presented with this randomized number directly prior to commencing the walking trial, to ensure that they had not already begun subtracting mentally. Once they had been presented with this randomized number, participants’ first verbalized response was the subtracted target value (i.e., first verbalization of 93 if the randomized number presented was 100). Participants were instructed to allocate an equal amount of attention towards both the walking and the arithmetic task. Participants completed eight blocks of two walking trials (16 trials total). Each experimental block consisted of one Baseline and one Dual-Task trial, presented in a randomized order. The walking route was rearranged after each block, with each pattern presented twice. Pattern presentation was randomized. The protocol was split into two halves, with a 20-minute mid-point break following the completion of block 4. Each half featured one-block of each of the four non-linear walking routes.

After each trial, participants completed the Rating Scale of Mental Effort (RSME; [39]). The RSME was used as a manipulation check, to measure perceived mental effort, in order to ensure that the cognitive load manipulation was successful in increasing cognitive demand. The RSME was presented as a single continuum scale ranging from 0 to 150, with nine validated reference points along the scale (e.g., “Absolutely No Effort”, “Some Effort”, “Extreme Effort”, etc.). Researchers have demonstrated that the scale provides a valid and reliable indicator of mental effort [40].

### Reinvestment

The Movement Specific Reinvestment Scale (MSRS) [41] was used to assess participants’ trait-reinvestment. This 10-item questionnaire consists of two 5-item subscales: conscious motor processing (trait-CMP; e.g., “I am always trying to think about my movements when I carry them out”) and movement self-consciousness (trait-MSC; e.g., “I’m self-conscious about the way I look when I am moving”). Items are rated on a 5-point Likert scale (0 = extremely uncharacteristic; 4 = extremely characteristic). Total MSRS scores range from 0–40, with higher scores reflecting a higher propensity for reinvestment. Both subscales have good internal validity and test-retest reliability [41]. Participants completed the MSRS once, during a 20-minute mid-point break (following the completion of block 4).

### Dual-Task Assessments

To quantify participants’ ability to execute two tasks concurrently, we calculated dual-task costs (DTCs) according to the customary formula [42]:

DTC(%)=100*(single−taskscore—dual−taskscore)/single−taskscore

Thus, higher DTCs reflect poorer performance under dual-task conditions.

#### Cognitive DTCs

Cognitive performance was defined as the number of correct arithmetic calculations verbalized. Dual-task scores were calculated during trials where participants performed the cognitive task while walking (Dual-Task trials). Single-task scores were calculated while participants performed the cognitive task from a seated position behind the screen. During the single-task condition, participants were given 30 seconds to subtract as many times as possible in 7s from a randomized number. The number of correct digits verbalized during Dual-Task trials were then compared to those verbalized during a proportional period of time during single-task. For example, if a participant completed a Dual-Task walking trial in 15 seconds, the number of correct arithmetic calculations verbalized here was compared to the number verbalized in the first 15 seconds during single-task. Verbalizations were recorded from the eye-tracker’s integrated microphone, and then analyzed in Adobe Premiere Pro CC (Adobe Systems, San Jose, CA).

#### Motor DTCs

Performance on two separate motor tasks was calculated: (1) Time to complete the walking trial (defined as the time taken in seconds between the sternum marker crossing over the threshold of the first wooden block of the walking path and then crossing from the last wooden block onto the second ramp following the completion of the trial (see Fig 1)); and (2) Absolute stepping accuracy (total distance in mm between the mid-foot marker and the middle of the precision stepping accuracy target, regardless of axis). Prior to the commencement of the first trial in each block of trials, a static trial was recorded to identify the coordinates of both stepping targets. This procedure consisted of a reflective marker being placed in the middle of each target; the coordinates of which were then later used as a reference against the position of the mid-foot markers during each walking trial. As markers were placed in the middle of both feet, the co-ordinates of the mid-foot marker nearest the middle of the precision stepping accuracy target was used to calculate stepping accuracy. Motor performance data were processed using a low pass Butterworth filter at 5 Hz and then analyzed using custom algorithms in MATLAB version 7.11 (MathWorks, Natick, MA). For both variables, single-task performance was calculated during trials of Baseline walking, while dual-task scores were calculated during trials where participants performed the cognitive task while walking (Dual-Task trials).

### Gaze Behavior

**Fig 2:**
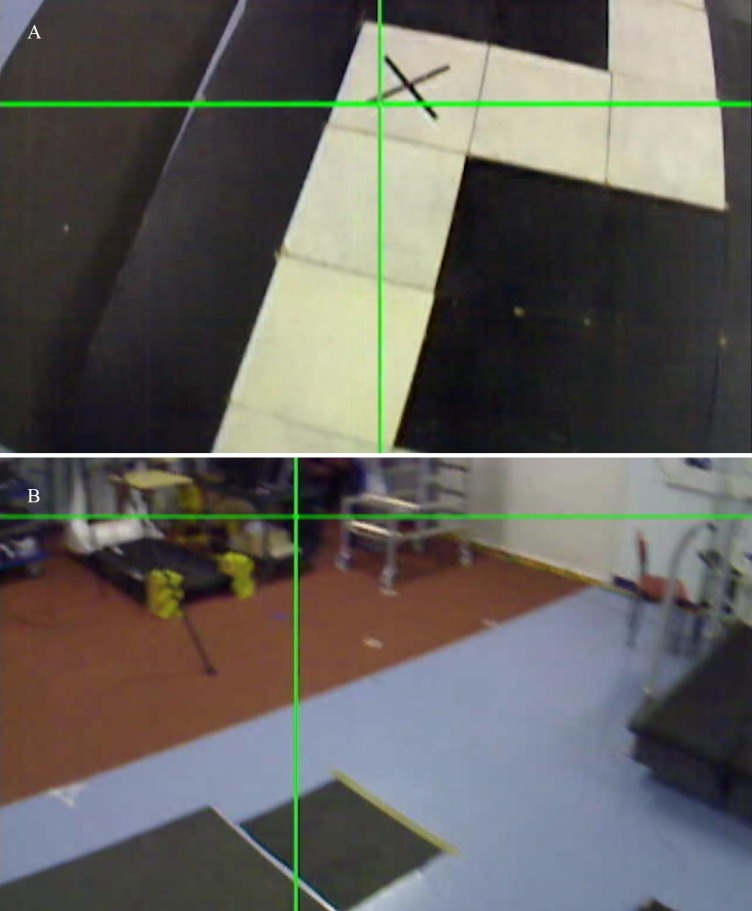
**a.** An example of a task-relevant ‘inside’ fixation, whereby the participant fixates on an area within their walking path. **b.** An example of a task-irrelevant ‘outside’ fixation, whereby the participant fixates on an area outside of their walking path.

Fixations were defined as a gaze that endured on a single location (≤ 1° visual angle) for 100 ms or longer [43]. It was reasoned that participants would need to fixate on both the white and black blocks within the walking path, as well as the ramp leading to, and from, the walkway, in order to acquire the relevant visuospatial information about their walking path. While black blocks were not part of the walking path, in that participants were instructed to avoid walking on these, participants still needed to acquire relevant visuospatial information about the location of these blocks in order to avoid stepping onto them; much in the same way that participants switch their gaze between both areas of the walking path on which they wish to step, and those which they wish to avoid, during obstacle avoidance [13]. However, other areas of the surrounding environment, such as the laboratory floor beyond the walking path or the laboratory walls, were deemed to contain no visual information necessary to aid the completion of the walking task. Therefore, fixations were classified as either task-relevant ‘inside’ fixations (any area of the environment necessary for safely navigating the walkway: the first and second ramp, and any white or black stepping block within the walking path (Fig 2A)) or task-irrelevant ‘outside’ fixations (any area of the surrounding environment that was not either a ramp, or white or black stepping block (Fig 2B)). Trials in which the point of gaze crosshair disappeared for the duration of three frames (100 ms) or more were discarded. Participants with 50% or greater trial-discard rate were excluded from all analyses. This procedure resulted in 3 participants being excluded from the analyses. Gaze data was analyzed between the point when participants stepped from behind the screen and initiated gait towards the first ramp, and the point when participants stepped from the final block of the walkway onto the second ramp. As the Motion Analysis system was not able to begin capturing data until participants stepped from the first ramp onto the walking path, we used the ASL eye-tracking videos to identify these points. As participants’ heads were pitched down at an angle that also captured their feet during the approach of the second ramp, this allowed for a reliable visual inspection of the frame in which the foot contacted the ramp.

### Temporal Relationship between Cognitive Load and Visual Search

**Fig 3:**
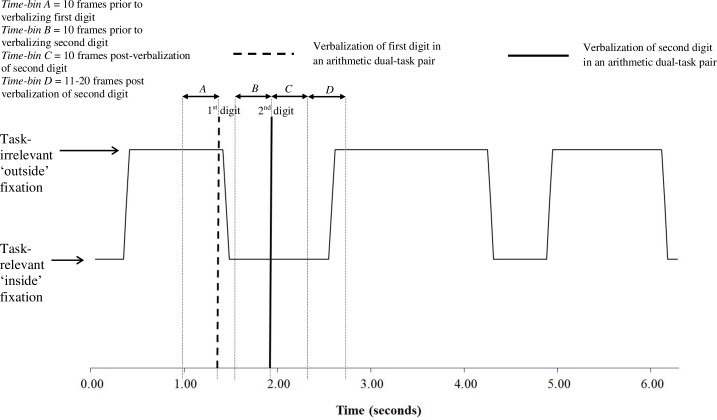
Schematic example of the time-bins utilized to investigate the temporal relationship between cognitive load and visual search.

While it was hypothesized that individuals would adopt a less-variable pattern of visual search under conditions of Cognitive Load, preliminary analysis revealed a very different pattern of behavior. Instead of reducing the variability of their visual search and dwelling on single points for longer periods of time, participants frequently disengaged their gaze from the walking path altogether to fixate on task-irrelevant ‘outside’ areas. Due to the unforeseen changes in visual search behaviour described above, we conducted a supplementary analysis evaluating the temporal interaction between task-irrelevant fixations and the verbalizations involved in performing the cognitive secondary task. For this temporal analysis, patterns of visual search were compared in four separate temporal time-bins (Fig 3): (1) 10 frames (330ms) prior to verbalizing the first digit in an arithmetic dual-task pair; (2) 10 frames prior to verbalizing the second digit in an arithmetic pair; (3) 10 frames post-verbalization of the second digit in an arithmetic pair, and; (4) 11–20 frames post verbalization of the second digit in an arithmetic pair.

Both gaze data and audio verbalizations were analyzed frame-by-frame using Adobe Premiere Pro CC (Adobe Systems, San Jose, CA). Ten-frame windows were selected to allow for the classification of distinct, non-overlapping time-bins. Presumably, cognitive processing would be required in both time-bins immediately prior to verbalizing both digits. However, after the verbalization of the second digit, we presumed that the necessary processing for this arithmetic pair was complete, allowing participants to redirect cognitive resources towards visually scanning their walking path. It was unfeasible to include a post-verbalization time-bin for the first digit in an arithmetic pair because, in several participants, the verbalization of both digits occurred in such quick succession that the two time-bins (following the first digit, and preceding the second) would often overlap. Therefore, the second digit of the arithmetic pair was identified as containing the most appropriate time-bins for a post-verbalization comparison, and patterns of visual search in a time-bin post verbalization of the first digit were not investigated. The percentage of time spent fixating task-irrelevant areas was calculated for each of the four time-bins detailed above.

### Statistical Analysis

#### Manipulation check

A paired-samples t-test was used to determine whether the Cognitive Load manipulation was successful in increasing cognitive demand, as determined by RSME scores. Effect size is reported as Cohen’s *d*.

#### DTCs

Separate paired-samples t-tests were used to determine whether there was a significant decrease in raw performance scores for either the cognitive task or the motor task (stepping accuracy) during Dual-Task trials, when compared to single-task. A Wilcoxon test was used to determine whether there was a significant decrease in raw performance scores for time to complete the walking trial during Dual-Task trials. The use of a non-parametric test was deemed necessary here and elsewhere in the paper if data were non-normally distributed. Separate paired-samples t-tests were then used to determine whether any DTCs observed for either the cognitive task or the motor task (stepping accuracy) were significant, when compared to zero (which represented identical Single- and Dual-Task performance). A Wilcoxon test was used to determine whether any DTCs observed for time to complete the walking trial were significant, when compared to zero. Effect size is reported as Cohen’s *d*, unless the assumption of normality is violated, whereby effect size is reported as *r* = *Z*/√*N* [44].

#### Gaze behavior

A paired-samples t-test was used to investigate the effect of Cognitive Load on the number of task-relevant ‘inside’ fixations. These data were normalized to trial length, with the number of fixations presented as the average number of fixations per second. Separate Wilcoxon’s tests were used to investigate the effect of Cognitive Load on the duration (as a percentage of overall fixation durations) of task-relevant ‘inside’ fixations, and on both the number (/per second) and duration (as a percentage of overall fixation durations) of task-irrelevant ‘outside’ fixations, as these data were non-normally distributed.

#### Temporal relationship between cognitive load and visual search

A repeated measures ANOVA was used to investigate the percentage of time spent fixating task-irrelevant areas during each temporal time-bin. Effect size is reported as partial eta squared (*ƞp*^*2*^). Any significant effects were followed up by pairwise comparisons with Bonferroni adjustments.

#### Correlations

Separate bivariate correlations were run between three measures of trait-reinvestment (trait-MSRS; trait-CMP; trait-MSC) and each of the aforementioned variables. As some variables of gaze data were not normally distributed (the duration of task-relevant ‘inside’ fixations for Baseline trials; the number of task-irrelevant ‘outside’ fixations for Baseline trials, and; the duration of task-irrelevant ‘outside’ fixations for Baseline trials), Spearman’s correlation were used for these comparisons. All other data were analyzed using Pearson’s correlation.

## Results

### Manipulation check

Participants reported significantly higher levels of mental effort during Dual-Task trials (*M* = 53.24, *SD* = 22.25), compared to Baseline (*M* = 10.21, *SD* = 6.44), *t*(10) = -7.45, *p* < .001, *d* = 2.63. There were no significant correlations between RSME scores and any measures of trait-reinvestment (trait-MSRS; trait-CMP; trait-MSC).

### Dual Task Assessments

#### Cognitive DTCs

**Fig 4:**
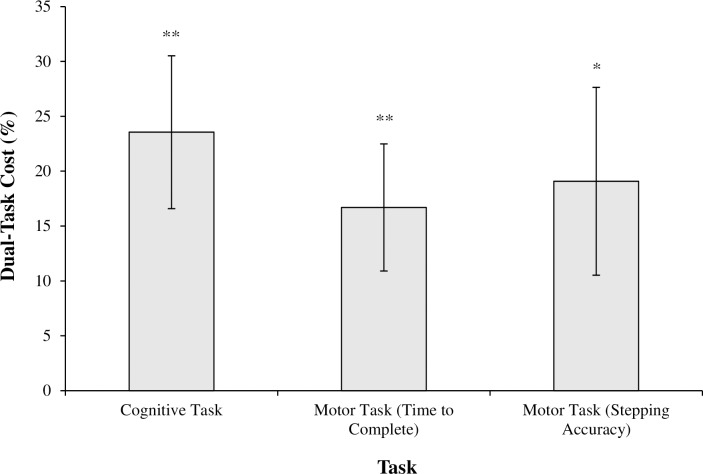
Dual-task costs (as a percentage decrease in performance compared to single-task performance) (mean ± standard error of the mean), * *p* < .05, ** *p* < .01.

Participants verbalized significantly fewer correct arithmetic calculations when completing the cognitive task while walking (*M* = 3.70, *SD* = 1.41), compared to when sitting (*M* = 4.91, *SD* = 1.45), *t*(10) = -3.99, *p* < .01, *d* = 0.85, with these DTCs (Fig 4) being significant, *t*(10) = -3.38, *p* < .01, *d* = 1.44. There were no significant correlations between cognitive DTCs and any measures of trait-reinvestment (trait-MSRS; trait-CMP; trait-MSC).

#### Motor DTCs

Participants took significantly longer to traverse the walkway (Single-Task, *M* = 4.58 seconds, *SD* = 0.95; Dual-Task, *M* = 5.45 seconds, *SD* = 1.95), *Z* = -2.93, *p* < .01, *r* = 0.88, and had poorer stepping accuracy (Single-Task, *M* = 58.83mm, *SD* = 35.54; Dual-Task, *M* = 68.07mm, *SD* = 37.95) when walking while simultaneously processing the cognitive dual-task, *t*(10) = -3.16, *p* < .01, *d* = 0.25, compared to when just walking. These DTCs were significant for both time to complete the walking trial, *Z* = -2.93, *p* < .01, *r* = 0.88, and absolute stepping accuracy, *t*(10) = -2.28, *p* < .05, *d* = 0.95. There were no significant correlations between motor DTCs and any measures of trait-reinvestment (trait-MSRS; trait-CMP; trait-MSC). The data are presented in Fig 4.

### Gaze Behavior

#### Task-relevant ‘inside’ fixations

**Fig 5:**
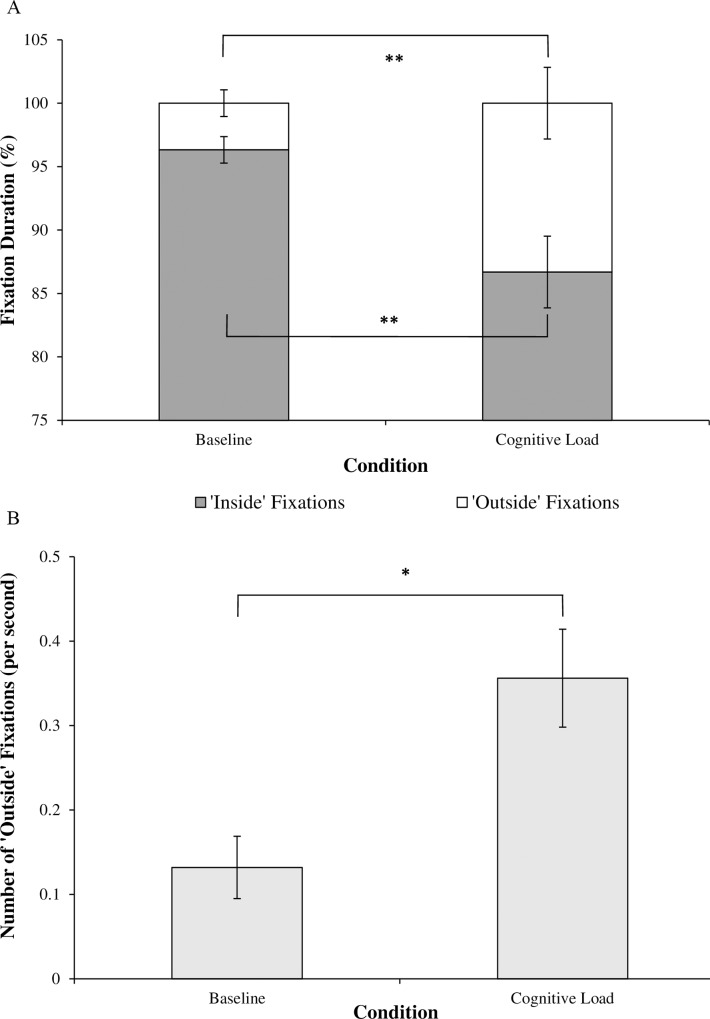
**a.** Duration (as a percentage of overall fixation durations) of task-relevant ‘inside’ and task-irrelevant ‘outside’ fixations under conditions of Cognitive Load (mean ± standard error of the mean), ** *p* < .01. **b.** Number of task-irrelevant ‘outside’ fixations under conditions of Cognitive Load (mean ± standard error of the mean), ** *p* < .05.

There was no significant effect of Cognitive Load on the number of task-relevant ‘inside’ fixations, *t*(10) = 1.38, *p* > .05, *r* = 0.34. However, there was a significant effect of Cognitive Load on the duration (as a percentage of overall fixation durations) of task-relevant ‘inside’ fixations, *Z* = -2.58, *p* < .01, *r* = 0.78. Participants spent significantly less time fixating on task-relevant ‘inside’ areas under conditions of Cognitive Load, when compared to Baseline. These data are presented in Fig 5A. This finding indicates that while participants did not differ in the number of task-relevant ‘inside’ fixations made under increased cognitive load, these fixations were of a shorter duration. Trait-MSC scores were negatively correlated with duration of time spent fixating on task-relevant ‘inside’ areas under conditions of Cognitive Load (*r* = -.71, *p* < .01), indicating that this reduction in task-relevant fixation durations was driven by high-trait-MSC individuals.

#### Task-irrelevant ‘outside’ fixations

There was a significant effect of Cognitive Load on the number, *Z* = -2.49, *p* < .05, *r* = 0.75, and duration (as a percentage of overall fixation durations) of task-irrelevant ‘outside’ fixations, *Z* = -2.58, *p* < .01, *r* = 0.78. Participants fixated ‘outside’ the walking path more often, and for longer durations of time under conditions of Cognitive Load, when compared to Baseline. These data are presented in Fig 5B. Trait-MSC scores were positively correlated with both the number (*r* = .69, *p* < .05) and duration of task-irrelevant ‘outside’ fixations under Cognitive Load (*r* = .71, *p* < .01), indicating that high-trait-MSC was associated with longer and more frequent fixations on task-irrelevant ‘outside’ areas under high cognitive load.

### Temporal Relationship between Cognitive Load and Visual Search

**Fig 6:**
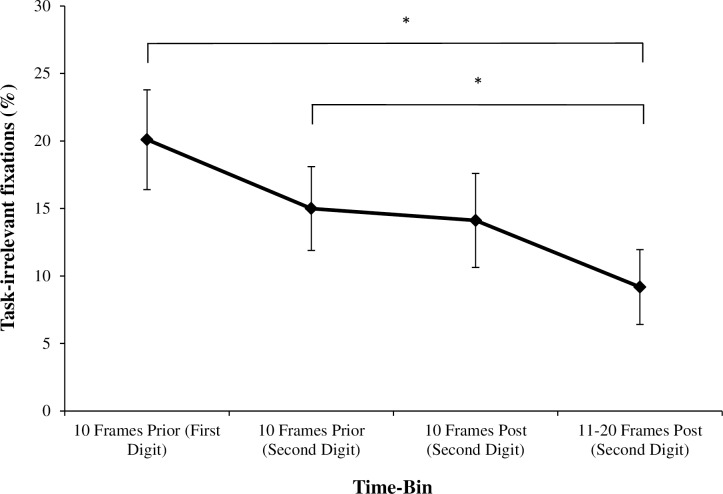
Percentage of time spent fixating task-irrelevant areas (as a %) during different time-bins (mean ± standard error of the mean), * *p* < .05.

There was a significant effect of Calculation Time-Bin on the amount of time spent fixating on ‘outside’ task irrelevant areas, *F*(3,30) = 6.97, *p* < .01, *ƞ*_*p*_^*2*^ = 0.41. Bonferonni post-hoc tests revealed that individuals spent significantly more time fixating task-irrelevant areas in the 10-frames prior to verbalizing both the first (*p* < .05) and second digit of an arithmetic dual-task pair (*p* < .05), when compared to 11-20-frames post second digit verbalization (Fig 6).

**Fig 7:**
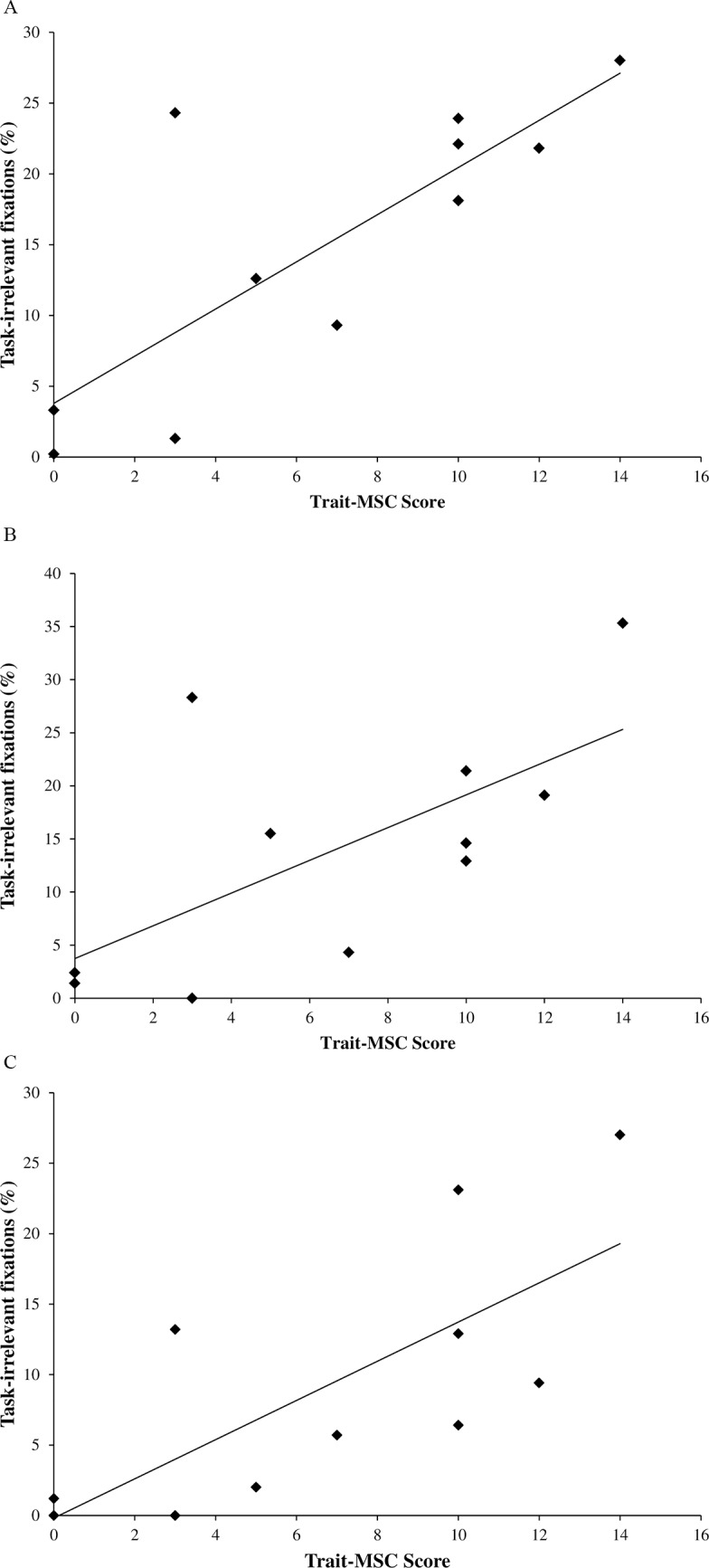
**a.** Correlation between trait-MSC and mean percentage of time spent fixating task-irrelevant areas in the 10 frames prior to verbalizing the second dual-task arithmetic value. **b.** Correlation between trait-MSC and mean percentage of time spent fixating task-irrelevant areas in the 10 frames post second value verbalization. **c.** Correlation between trait-MSC and mean percentage of time spent fixating task-irrelevant areas in the 11–20 frames post second value verbalization.

There were no significant correlations between the time spent fixating on ‘outside’ task irrelevant areas in the 10-frames prior to verbalizing the first digit and any measures of trait-reinvestment (trait-MSRS; trait-CMP; trait-MSC). However, as illustrated in Fig 7, trait-MSC was positively correlated with all three other time-bins. These included: time spent fixating on ‘outside’ task irrelevant areas in the 10-frames prior to verbalizing the second digit of an arithmetic dual-task pair (*r* = .80, *p* < .01); time spent fixating task-irrelevant areas in the 10-frames post second digit verbalization (*r* = .65, *p* < .05), and; time spent fixating task-irrelevant areas in the 11-20-frames post second digit verbalization (*r* = .73, *p* < .01). The lack of significant correlation between trait-MSC scores and the time spent fixating on ‘outside’ task irrelevant areas in the 10-frames prior to verbalizing the first digit in the dual-task calculation pair indicates that all participants initially prioritized the cognitive task over maintaining an effective pattern of visual search behaviors. However, in the 10-frames prior to verbalizing the second digit in the dual-task calculation pair, low trait MS-C individuals had already begun to reallocate attention towards feedforward planning of how to negotiate the walking path, whereas high trait MS-C individuals continued to ‘gaze into thin-air’ at least until 20-frames following the verbalization of the second digit in the dual-task arithmetic pair.

## Discussion

We examined whether increased cognitive load impacted on visual search behaviors during locomotion. Specifically, we examined whether the previously detailed less-variable patterns of visual search behaviors observed in high-risk older adults (i.e., focusing on the initial target for longer durations of time and failing to fixate on subsequent upcoming constraints) could be induced in a healthy younger adult cohort walking while simultaneously carrying out a cognitive dual-task.

Our results suggest that walking under increased cognitive load impaired individuals’ ability to maintain effective visual search. However, the pattern of behavior observed was different to that which was expected. Instead of adopting a less-variable pattern of gaze behavior (as described by [13]), younger adults appeared to disengage visual attention from their walking environment; a behaviour that might be termed ‘gazing into thin air’. As illustrated in Fig 5, while walking under cognitive load participants fixated on task-irrelevant areas ‘outside’ the walking path more often, and for longer durations of time, and fixated on task-relevant areas ‘inside’ the walkway for shorter durations.

It has been suggested that the reductions in visual search observed in high-risk older adults may be caused by a form of conditioning, in which these individuals fail to effectively scan their environment as a result of being unable to retain the visual-spatial information that proactive visual search generates [16]. Therefore, it is possible that younger adults similarly disengaged from visually scanning their environment under conditions of Cognitive Load due to the reduced availability of cognitive resources necessary to store this ‘spatial map’ of their surroundings in working memory. This latter suggestion is supported by research demonstrating how walking while simultaneously talking on a cell phone can cause ‘situational blindness’, whereby younger adults fail to perceive unusual objects along their walking path, such as a unicycling clown [34] or a tree with money attached to the leaves [35]. However, as gaze behavior was not measured in this previous research, it is difficult to assess whether this ‘situational blindness’ was in fact a consequence of individuals failing to visually scan their environment. For example, it is equally possible that participants’ visual search strategies were unchanged, and that cell phone usage merely disrupted the storing of this information (something supported by literature demonstrating reduced memory for objects during dual-task conditions of driving, even when the objects are directly fixated on [45,46]). As visuo-spatial memory was not assessed in the present experiment, in future researchers should look to investigate the relationship between cognitive load, gaze behavior, and the retention of the visual information acquired through proactive visual search.

These changes in visual search behavior under Cognitive Load were accompanied by significant increases in both time to complete the walking task and absolute stepping errors. Researchers have demonstrated that an absence of visual information during gait can result in both a more cautious walking pattern (i.e. slower speed, smaller steps) [47] and impaired stepping accuracy [11]. Therefore, it is entirely plausible that these observed reductions in motor performance under conditions of Cognitive Load may be a consequence of participants spending less time visually previewing their walking path and more time fixating on task-irrelevant areas ‘outside’ of the walkway. This proposal is supported by research demonstrating that dual-task-related declines in gait performance are more pronounced during walking tasks requiring greater visual processing and feedforward visual planning, such as obstacle avoidance [26]. However, this proposed relationship between cognitive load-induced inefficiencies in visual search and impaired gait performance needs to be explored in greater detail before a causal link can be established. Unfortunately, given the highly variable nature of the walking task utilized in the present research, we were unable to conduct an analysis to quantify the temporal relationship between gait and gaze behavior and establish a causal link. For example, as task-irrelevant ‘outside’ fixations occurred both on straight sections of the walkway and during turns, gait velocity would likely differ independent of gaze location.

Our results also demonstrate a clear temporal relationship between changes in visual search and the processing of a cognitive second task. As illustrated in Fig 6, participants spent significantly longer time periods ‘gazing into thin air’ in the time-bins directly preceding an arithmetic calculation (verbalization of both the first and second digit of an arithmetic calculation pair), when compared to the time-bins post-verbalization of the second digit. This finding suggests that, in regards to disrupting visual search, the two most attentionally demanding periods of the arithmetic calculation are in the 10-frames (330ms) prior to verbalizing either the first or the second value in the arithmetic calculation pair. These results indicate that younger adults disengaged visual attention from the walking path in order to prioritize the processing of information relevant to the cognitive secondary task. This observation suggests that acquiring and processing visual information during adaptive gait carries an attentional demand, which can be significantly disrupted under dual-task conditions.

From a working memory perspective (e.g., [48]), we might have predicted that the level of interference between the two tasks should have been minimal, because each depends on a different aspect of working memory. For example, the information obtained through visual search behavior is likely to be processed and stored within the visuo-spatial sketchpad (a system dedicated to maintaining and manipulating visuo-spatial information), while the arithmetic dual-task is likely to be processed within the phonological loop (a short-term buffer responsible for storing and processing verbal information) [48]. However, our results suggest that the two tasks may share common central processing resources. Acquiring visual information during adaptive gait requires online control of gaze behavior, with this information being monitored and updated [49,50]; two processes which require input from the central executive component of working memory [51]. In addition, researchers have demonstrated how loading the central executive impairs performance on a wide range of mental arithmetic tasks [52–56], suggesting that the central executive plays a role in even simple arithmetic calculations. Therefore, our results suggest that a form of structural interference occurred, with both tasks competing for common central executive resources, and participants disengaging from visually scanning their environment in order to prioritize the arithmetic calculation. According to this rationale, visual search during locomotion may be disrupted by the simultaneous processing of any task requiring central executive input, which may include anxiety-related processes [57,58] and conscious movement monitoring and control (i.e., reinvestment) [16].

Our paper is the first to indicate a relationship between trait-reinvestment and changes in visual search strategies. As predicted, the changes in visual search behaviors under cognitive load were the most pronounced in high-trait-reinvesters. We found that trait-MSC scores (a measurement of how self-conscious an individual is about the way they look when moving) were negatively correlated with the duration of time spent fixating on task-relevant ‘inside’ areas under Cognitive Load, and positively correlated with both the number and duration of task-irrelevant ‘outside’ fixations under Cognitive Load. We suggest that the aforementioned significant Baseline-Cognitive Load changes in visual search were driven by the gaze behavior of high-trait-reinvesters. As trait-MSC scores were not correlated with cognitive DTCs or mental-effort ratings, these alterations in gaze behavior were not merely caused by differences in participants’ ability to complete the cognitive dual-task. Previously, researchers have suggested that trait-reinvestment places greater cognitive demands upon the working memory of older adults [37]. Therefore, we speculate that trait-MSC-related processes (such as forming a visual representation of the way you move, or ruminating about the way you look when you move) may place demands on the central executive component of working memory. These results indicate that due to demands associated with maintaining an awareness of body movements, high-trait-MSC individuals disengaged from visually scanning the walking path in order to make the necessary cognitive resources available to perform the arithmetic task.

Our results also demonstrate that trait-reinvestment was related to temporal changes in visual search. While there was no significant correlation between trait-MSC scores and the time spent ‘gazing into thin air’ in the 10-frames prior to verbalizing the first digit of an arithmetic dual-task pair, strong, significant positive correlations were observed between trait-MSC scores and the time spent ‘gazing into thin air’ in: 1) the 10-frames prior to verbalizing the second digit of an arithmetic dual-task pair; 2) the 10-frames post second digit verbalization; and 3) the 11-20-frames post second digit verbalization. These correlations, in combination with the aforementioned temporal analysis data, suggest that, regardless of trait-reinvestment scores, younger adults in general experienced structural interference in the 10-frames prior to verbalizing the first digit of an arithmetic dual-task pair, resulting in a situation where they had to prioritize between visual search and the cognitive task. Participants then disengaged from visually scanning their walking path in order to prioritize this cognitive task. However, as illustrated in Fig 7, in the 10-frames prior to verbalizing the second value in the dual-task calculation pair, low-trait-MSC individuals began to reallocate attention towards the walking path. In contrast, high-trait-MSC individuals continued to ‘gaze into thin air’ up to 20-frames (660ms) following the verbalization of the second digit in the dual-task calculation. This finding suggests that trait-reinvestment disrupted the ability to re-engage visual attention towards the path following an incidence of ‘gazing into thin air’.

One of the roles of the central executive is to allocate attention between tasks [51]. Therefore, it is conceivable that demands placed on the central executive by reinvestment-related processes disrupted individuals’ abilities to switch attention between processing the cognitive dual-task and carrying out effective visual search. Young and colleagues [37] found higher levels of trait-reinvestment in older adults who ‘stopped walking when talking’; that is to say, individuals who were unable to effectively switch attention between two tasks (walking and answering a researcher’s question). If trait-reinvestment does disrupt the allocation of attention between tasks it is, therefore, possible that the previously detailed postural stiffening observed in high-trait-MSC younger adults under conditions of anxiety [59] may not relate to conscious movement control and the freezing of degrees of freedom associated with deliberate control of an automatic movement, as previously suggested [16]. This reinvestment-related stiffening may be a protective mechanism for stabilizing posture, so as to allow disengagement of attention from postural control for purposes of anxiety-related processing. Our results suggest that older adults with high levels of trait-reinvestment might be particularly susceptible to reductions in the efficiency of visually previewing an intended path when carrying out a concurrent task. This is particularly worrying, as reduced visual previewing is associated with increased frequency of gross stepping errors in this population [13,16]. However, as we only explored changes in visual search behaviors under cognitive load in a younger adult cohort, the results presented cannot be generalized to clinical populations at a high risk of falling (i.e., high-anxious older adults). For example, as the walking task utilized in the present research was one of both relative simplicity and low risk, it is possible that younger adults felt able to safely disengage from visually scanning their environment whilst maintaining balance. Published reports suggest that the degree to which older adult fallers prioritize a postural task over a cognitive dual-task is dependent upon perceived risk [60]. Consequently, in future, researchers should look to replicate these findings in older adults at a high risk of falling.

## Conclusion

Our results demonstrate that cognitive load impacted the visual search efficiency of younger adults during an adaptive gait task. However, the cognitive secondary task did not induce a pattern of visual search commonly observed in high-risk older adults. While walking under conditions of Cognitive Load, young adults fixated on task-irrelevant areas ‘outside’ the walking path more often, and for longer durations of time, and fixated on task-relevant areas ‘inside’ the walkway for shorter durations. These changes were most pronounced in high-trait-MSC individuals, presumably because reinvestment-related processes placed an additional cognitive demand upon working memory. These increased task-irrelevant ‘outside’ fixations were accompanied by both slower walking task completion rates and greater gross stepping errors, indicating that these changes in visual search negatively impacted gait performance. The findings suggest that attention is important for the maintenance of effective gaze behaviors, supporting previous claims that aforementioned maladaptive changes in visual search observed in high-risk older adults may be a consequence of inefficiencies within attentional processing [16]. Identifying the underlying attentional processes that disrupt effective gaze behaviour during locomotion is an essential step in the development of rehabilitation, with this information allowing for the emergence of empirically grounded falls-prevention tools.

## Supporting Information

S1 Dataset Dataset. (SAV)

## References

[1] Lord SR, Dayhew J. Visual risk factors for falls in older people. J Am Geriatr Soc. 2001; 49: 508–15. 11380741 10.1046/j.1532-5415.2001.49107.x

[2] Lord SR. Visual risk factors for falls in older people. Age Ageing. 2006; 35 Suppl 2: 42–5. 16926203 10.1093/ageing/afl085

[3] Beurskens R, Bock O. Age-related decline of peripheral visual processing: The role of eye movements. Exp Brain Res. 2012; 217: 117–24. 10.1007/s00221-011-2978-3 22179529 PMC3279647

[4] Białoszewski D, Słupik A, Lewczuk E, Gotlib J, Mosiołek A, Mierzwińska A. Incidence of falls and their effect on mobility of individuals over 65 years of age relative to their place of residence. Ortop Traumatol Rehabil. 2008; 10: 441–8. 19043351

[5] Tinetti ME, Williams CS. Falls, injuries due to falls, and the risk of admission to a nursing home. N Engl J Med. 1997; 337: 1279–84. 10.1056/NEJM199710303371806 9345078

[6] NICE Guidelines. Falls: The Assessment and Prevention of Falls in Older People. 2013. 25506960

[7] National Center for Injury Prevention and Control. Web-Based Injury Statistics Query and Reporting System (WISQARS). 2013.

[8] Hollands MA, Marple-Horvat DE, Henkes S, Rowan AK. Human Eye Movements During Visually Guided Stepping. J Mot Behav. 1995; 27: 155–63. 10.1080/00222895.1995.9941707 12736124

[9] Hollands MA, Marple-Horvat DE. Visually guided stepping under conditions of step cycle-related denial of visual information. Exp brain Res. 1996; 109: 343–56. 8738381 10.1007/BF00231792

[10] Hollands MA, Marple-Horvat DE. Coordination of eye and leg movements during visually guided stepping. J Mot Behav. 2001; 33: 205–16. 10.1080/00222890109603151 11404215

[11] Chapman GJ, Hollands MA. Age-related differences in stepping performance during step cycle-related removal of vision. Exp brain Res. 2006; 174: 613–21. 10.1007/s00221-006-0507-6 16733708

[12] Chapman GJ, Hollands MA. Evidence that older adult fallers prioritise the planning of future stepping actions over the accurate execution of ongoing steps during complex locomotor tasks. Gait Posture. 2007; 26: 59–67. 10.1016/j.gaitpost.2006.07.010 16939711

[13] Young WR, Wing AM, Hollands MA. Influences of state anxiety on gaze behavior and stepping accuracy in older adults during adaptive locomotion. Journals Gerontol—Ser B Psychol Sci Soc Sci. 2012; 67: 43–51. 10.1093/geronb/gbr074 21808071

[14] Young WR, Hollands MA. Newly acquired fear of falling leads to altered eye movement patterns and reduced stepping safety: A case study. PLoS One. 2007; 7: e49765. 10.1371/journal.pone.0049765 PMC3504096 23185432

[15] Zettel JL, Scovil CY, McIlroy WE, Maki BE. Gaze behavior governing balance recovery in an unfamiliar and complex environment. Neurosci Lett. 2007; 422: 207–12. 10.1016/j.neulet.2007.06.020 17611033

[16] Young WR, Williams AM. How fear of falling can increase fall-risk in older adults: Applying psychological theory to practical observations. Gait Posture. 2015; 41: 7–12. 10.1016/j.gaitpost.2014.09.006 25278464

[17] Young WR, Hollands MA. Can telling older adults where to look reduce falls? Evidence for a causal link between inappropriate visual sampling and suboptimal stepping performance. Exp brain Res. 2010; 204: 103–13. 10.1007/s00221-010-2300-9 20512484

[18] Al-Yahya E, Dawes H, Smith L, Dennis A, Howells K, Cockburn J. Cognitive motor interference while walking: A systematic review and meta-analysis. Neurosci Biobehav Rev. 2011; 35: 715–28. 10.1016/j.neubiorev.2010.08.008 20833198

[19] Yogev-seligmann G, Hausdorff JM, Giladi N. The role of executive function and attention in gait. Mov Disord. 2008; 23: 329–42. 10.1002/mds.21720 18058946 PMC2535903

[20] Muir SW, Gopaul K, Montero Odasso MM. The role of cognitive impairment in fall risk among older adults: A systematic review and meta-analysis. Age Ageing. 2012; 41: 299–308. 10.1093/ageing/afs012 22374645

[21] Schwebel DC, Stavrinos D, Byington KW, Davis T, Neal EE, De Jong D. Distraction and pedestrian safety: How talking on the phone, texting, and listening to music impact crossing the street. Accid Anal Prev. 2012; 45: 266–71. 10.1016/j.aap.2011.07.011 22269509 PMC3266515

[22] Stavrinos D, Byington KW, Schwebel DC. Distracted walking: Cell phones increase injury risk for college pedestrians. J Safety Res. 2011; 42: 101–7. 10.1016/j.jsr.2011.01.004 21569892

[23] Agmon M, Belza B, Nguyen HQ, Logsdon RG, Kelly VE. A systematic review of interventions conducted in clinical or community settings to improve dual-task postural control in older adults. Clin Interv Aging. 2014; 9: 477–92. 10.2147/CIA.S54978 24741296 PMC3970921

[24] Beurskens R, Bock O. Age-related Deficits of dual-task walking: A review. Neural Plast. 2012; Volume 2012: Article ID 131608 10.1155/2012/131608 PMC3403123 22848845

[25] Bock O. Dual-task costs while walking increase in old age for some, but not for other tasks: An experimental study of healthy young and elderly persons. J Neuroeng Rehabil. 2008; 5: 27 10.1186/1743-0003-5-27 19014544 PMC2596160

[26] Beurskens R, Bock O. Does the walking task matter? Influence of different walking conditions on dual-task performances in young and older persons. Hum Mov Sci. 2013; 32: 1456–66. 10.1016/j.humov.2013.07.013 24035208

[27] Lundin-Olsson L, Nyberg L, Gustafson Y. “Stops walking when talking” as a predictor of falls in elderly people. Lancet. 1997; 349: 617. 10.1016/S0140-6736(97)24009-2 9057736

[28] Muir-Hunter SW, Wittwer JE. Dual-task testing to predict falls in community-dwelling older adults: a systematic review. Physiotherapy. 2015; 102: 29–40. 10.1016/j.physio.2015.04.011 26390824

[29] Nagamatsu LS, Voss M, Neider MB, Gaspar JG, Handy TC, Kramer AF, et al Increased cognitive load leads to impaired mobility decisions in seniors at risk for falls. Psychol Aging. 2011; 26: 253–9. 10.1037/a0022929 21463063 PMC3123036

[30] Beauchet O, Annweiler C, Dubost V, Allali G, Kressig RW, Bridenbaugh S, et al (Stops walking when talking: A predictor of falls in older adults? Eur J Neurol. 2009; 16: 786–95. 10.1111/j.1468-1331.2009.02612.x 19473368

[31] Wong WL, Masters RSW, Maxwell JP, Abernethy AB. Reinvestment and falls in community-dwelling older adults. Neurorehabil Neural Repair. 2008; 22: 410–4. 10.1177/1545968307313510 18334603

[32] Jackson RC, Ashford KJ, Norsworthy G. Attentional focus, dispositional reinvestment, and skilled motor performance under pressure. J Sport Exerc Psychol. 2006; 28: 49–68.

[33] Masters RSW, Maxwell J. The theory of reinvestment. Int Rev Sport Exerc Psychol. 2008; 1: 160–83.

[34] Hyman IE, Boss SM, Wise BM, McKenzie KE, Caggiano JM. Did you see the unicycling clown? Inattentional blindness while walking and talking on a cell phone. Appl Cogn Psychol. 2009; 24: 597–607.

[35] Hyman IE, Sarb BA, Wise-Swanson BM. Failure to see money on a tree: inattentional blindness for objects that guided behavior. Front Psychol. 2014; 5: Article 356. 10.3389/fpsyg.2014.00356 PMC4005951 24795686

[36] Uiga L, Capio CM, Wong TWL, Wilson MR, Masters RSW. Movement specific reinvestment and allocation of attention by older adults during walking. Cogn Process. 2015; 16(S1): 421–4. 26233524 10.1007/s10339-015-0685-x

[37] Young WR, Olonilua M, Masters RSW, Dimitriadis S, Mark Williams A. Examining links between anxiety, reinvestment and walking when talking by older adults during adaptive gait. Exp brain Res. 2016; 234: 161–72. 10.1007/s00221-015-4445-z 26403296 PMC4713710

[38] Young WR, Hollands MA. Evidence for age-related decline in visuomotor function and reactive stepping adjustments. Gait Posture. 2012; 36: 477–81. 10.1016/j.gaitpost.2012.04.009 22609043

[39] Zijlstra FRH. Efficiency in Work Behaviour: A Design Approach For Modern Tools TU Delft, Delft University of Technology; 1993.

[40] Veltman JA, Gaillard AWK. Physiological indices of workload in a simulated flight task. Biol Psychol. 1996; 42: 323–42. 8652751 10.1016/0301-0511(95)05165-1

[41] Masters RSW, Eves FF, Maxwell JP. Development of a movement specific reinvestment scale. In: Proceedings of the ISSP 11th World Congress of Sport Psychology, Sydney, Australia; 2005.

[42] McDowd JM. The effects of age and extended practice on divided attention Performance. J Gerontol. 1986; 41: 764–9. 3772053 10.1093/geronj/41.6.764

[43] Patla AE, Vickers JN. Where and when do we look as we approach and step over an obstacle in the travel path? Neuroreport. 1997; 8: 3661–5. 9427347 10.1097/00001756-199712010-00002

[44] Fritz CO, Morris PE, Richler JJ. Effect size estimates: Current use, calculations, and interpretation. J Exp Psychol Gen. 2012; 141: 2–18. 10.1037/a0024338 21823805

[45] Strayer DL, Drews F, Johnston W. Cell phone-induced failures of visual attention during simulated driving. J Exp Psychol Appl. 2003; 9: 23–32. 12710835 10.1037/1076-898x.9.1.23

[46] Strayer DL, Cooper JM, Drews F. What do drivers fail to see when conversing on a cell phone? Proc Hum Factors Ergon Soc Annu Meet. 2004; 48: 2213–7.

[47] Terrier P, Deriaz O, Reynard F. Role of vision in gait stabilization: Local dynamic stability in treadmill walking while blindfolded. J Neurol Sci. 2013; 333: e570–1.

[48] Baddeley AD, Hitch GJ. Working memory In: Bower G.H. (Ed.), The Psychology of Learning and Motivation: Advances in Research and Theory. New York: Academic Press; 1974 pp. 47–89.

[49] Higuchi T. Visuomotor control of human adaptive locomotion: Understanding the anticipatory nature. Front Psychol. 2013; 4: 1–9. 23720647 10.3389/fpsyg.2013.00277 PMC3655271

[50] Uiga L, Cheng KC, Wilson MR, Masters RSW, Capio CM. Acquiring visual information for locomotion by older adults: A systematic review. Ageing Res Rev. 2015; 20: 24–34. 10.1016/j.arr.2014.12.005 25576650

[51] Henry L. The Development of Working Memory in Children SAGE Publications; 2011.

[52] De Rammelaere S, Stuyven E, Vandierendonck A. The contribution of working memory resources in the verification of simple mental arithmetic sums. Psychol Res. 1999; 62: 72–7.

[53] De Rammelaere S, Stuyven E, Vandierendonck A. Verifying simple arithmetic sums and products: Are the phonological loop and the central executive involved? Mem Cognit. 2001; 29: 267–73. 11352209 10.3758/bf03194920

[54] De Rammelaere S, Vandierendonck A. Are executive processes used to solve simple arithmetic production tasks? Curr Psychol Lett. 2001; 2: 79–89

[55] Seitz K, Schumann-Hengsteler R. Mental multiplication and working memory. Eur J Cogn Psychol. 2000; 12: 552–70.

[56] Imbo I, Vandierendonck A. The role of phonological and executive working memory resources in simple arithmetic strategies. Eur J Cogn Psychol. 2007; 19: 910–933

[57] Eysenck MW, Derakshan N, Santos R, Calvo MG. Anxiety and cognitive performance: attentional control theory. Emotion. 2007; 7: 336–53. 10.1037/1528-3542.7.2.336 17516812

[58] Derakshan N, Eysenck MW. Anxiety, processing efficiency, and cognitive performance: New developments from attentional control theory. Eur Psychol. 2009; 14: 168–76.

[59] Zaback M, Cleworth TW, Carpenter MG, Adkin AL. Personality traits and individual differences predict threat-induced changes in postural control. Hum Mov Sci. 2015; 40: 393–409. 10.1016/j.humov.2015.01.015 25687665

[60] Muhaidat J, Kerr A, Evans JJ, Skelton D. Exploring gait-related dual task tests in community-dwelling fallers and non-faller: a pilot study. Physiother Theory Pr. 2013; 29: 351–70. 10.3109/09593985.2012.752056 23289962

